# Cementless Primary Stems in Revision Hip Arthroplasty: A Narrative Review

**DOI:** 10.3390/jcm13020604

**Published:** 2024-01-21

**Authors:** Francesco Castagnini, Francesco Pardo, Stefano Lucchini, Marco Rotini, Bruno Cavalieri, Mattia Dalla Rosa, Stefano Vitacca, Alberto Di Martino, Cesare Faldini, Francesco Traina

**Affiliations:** 1 Ortopedia-Traumatologia e Chirurgia Protesica e dei Reimpianti d’Anca e di Ginocchio, IRCCS Istituto Ortopedico Rizzoli, Via Pupilli 1, 40136 Bologna, Italy; francesco.pardo@ior.it (F.P.); stefano.lucchini@ior.it (S.L.); marco.rotini@ior.it (M.R.); bruno.cavalieri@ior.it (B.C.); mattia.dallarosa@ior.it (M.D.R.); stefano.vitacca@ior.it (S.V.); francesco.traina@ior.it (F.T.); 2 1^st^ Orthopedics and Traumatology Department, IRCCS Istituto Ortopedico Rizzoli, Via Pupilli 1, 40136 Bologna, Italy; albertocorrado.dimartino@ior.it (A.D.M.); cesare.faldini@ior.it (C.F.); 3 Department of Biomedical and Neuromotor Sciences—DIBINEM, University of Bologna, 40126 Bologna, Italy

**Keywords:** short stem, revision, canal flare, conservative, primary, de-escalation, conventional, tapered, downsizing

## Abstract

Cementless primary stems in revision hip arthroplasties may be conservative options to preserve bone stock and provide adequate reconstruction of the hip biomechanics. However, there is still little evidence about indications, limitations, and outcomes. This narrative review showed that conventional standard stems were adopted in different revision settings, up to Paprosky IIIA grade bone defects. In cases of acceptable metaphyseal bone stock, when a scratch fit of at least 4 cm can be achieved, a conventional cementless stem may be an adequate solution. Mid-term clinical and radiographic outcomes and survival rates were similar to long revision stems, whereas complications, surgical time, and costs were lower among conventional stems. However, unsuitable contexts for conventional stems included canal diameters larger than 18 mm and failed revision stems with cortical weakening. Even short stems can be considered in revisions, in order to preserve bone stock and stay proximal to femoral remodeling zones and bone/cement plugs. Short stems were successfully adopted up to Paprosky IIIA bone defects, achieving mid-term survival rates not inferior to long revision stems. Ageing, osteoporosis, and intraoperative femoral fractures were the main negative prognostic factors. In very select cases, a downsizing technique (from longer to shorter stems) may be adopted to simplify the procedure and reduce complications.

## 1. Introduction

According to some national registries, the estimated incidence of revision hip arthroplasty procedures has been around 10.2–22.3/100.000 adults per year for the last 15 years [1,2]. The mean age at revision is now set at 71 ± 11.3 years, with a sizable involvement of younger patients [1,2]. Out of all the revisions, femoral components are revised in 30–40% of the cases [1,3]. Thus, femoral stem revisions in active patients younger than 65 years of age are not uncommon procedures, and appropriate surgical strategies should be developed to address this relevant condition.

The femoral revision strategy adopts a wide portfolio of implants, with many possible techniques correlated to the underlying femoral bone defects and morphological alterations [4]. After the modest outcomes of cemented stems, a cementless solution was generally preferred, with the aim to provide a biological long-term fixation and the possible restoration of bone stock [4]. Traditionally, in revisions using cementless stems, the femoral component was required to extend distally over the bone defect, by at least twice the cortical diameter, or to engage 4–6 cm of the diaphyseal viable bone stock [4,5]. The most commonly adopted cementless revision stems were extensively porous-coated stems and tapered stems (modular and monoblock) [4]. Extensively porous-coated stems provided encouraging outcomes, but showed some notable limitations due to stress shielding and limited effectiveness in cases involving severe defects [4]. On the contrary, tapered stems are the current workhorse for complex femoral revisions, in the absence of severe damage to the isthmus [4]. Tapered stems require a 4–6 cm long diaphyseal fixation, with splines providing rotational stability [4,6]. Tapered stems demonstrated a general re-revision rate of 5.1%, with a survival rate ranging from 92% to 98.5% for non-modular solutions, and from 75% to 98% for modular options [6]. 

However, the widespread adoption of short stems in primary total hip arthroplasty, the increasing percentage of young patients with replaced hips, and the consequential need for revisions in young patients make conservative approaches in revision hips a desirable solution in order to avoid unnecessary and precocious femoral escalation. Conservative strategies for femoral component revisions have been developed to be “as proximal as possible and as distal as necessary”, with the aim to spare as much bone stock as possible for eventual future revisions, and to avoid intraoperative complications due to diaphyseal-engaging stems and distal bone stock violations [5,7]. While a primary cemented stem is a possible effective conservative solution in cases of previously cemented components (cement-in-cement) or minimal bone stock loss, cementless stem revisions have been gaining popularity and are generally preferred for their biological fixations and presumably better long-term outcomes, as well as surgeon preference [4,8]. There are two possible conservative options for cementless primary stems: conventional stems and short stems [5]. To date, there is a conspicuous literature about the use of cementless primary standard stems for femoral component revisions [5]. There is also a growing body of literature describing the first outcomes of short stems in revision hips [5]. However, the low quality of the papers, with mixed case series and different stem geometries adopted in various revision settings, precludes any definitive conclusions about the correct indications and the expected outcomes of cementless primary stems in revisions [5]. In particular, there is a lack of clear guidelines, perspectives, outcomes, and limitations for conservative approaches [5]. A narrative review was conducted to explore the indications, the limitations, and the outcomes of cementless primary stems (standard and short in length) in femoral revision hips.

## 2. Standard Stems

Lawrence et al. evaluated 83 revisions performed due to stem aseptic loosening at a minimum follow-up of 5 years. Different cementless primary stems were used: one stem was adopted in 77.1% of the cases (AML, DePuy, Warsaw, IN, USA: extensively coated stem with distal fixation) [9]. At a mean follow-up of 108 months, 23% of the hips underwent an additional operation, and 20% of them required a re-revision. There was a significant association between survival and canal filling. In 13% of the cases, there was no functional improvement after revision. A total of 44% of the cases returned to work. 

Moreland et al. reviewed 175 cementless femoral hip revision surgeries performed with a cementless primary stem (AML, DePuy, Warsaw, IN, USA) in femoral defects graded I (28.5%), II (52.8%), and III (18.8%), in accordance with Paprosky, at a mean follow-up of 5 years [10]. Femoral component re-revisions occurred in 4% of the cases. Stems achieved a valid bony ingrowth in 82.8% of the cases, especially when a good canal filling was achieved and there was no severe pre-operative bone stock deficiency. Severe stress shielding occurred in 7.6% of cases, due to pre-operative osteoporosis and larger diameter components. Relevant thigh pain occurred in 8% of the cases, and was more common in stable fibrous stems and osteoporotic femurs. 

Krishnamurthy et al. evaluated a series of 297 patients revised using a cementless primary stem at a minimum follow-up of 5 years (AML, DePuy, Warsaw, IN, USA) [11]. In the case series, 33% of the cases were graded III according to the Paprosky classification. Clinical outcomes were satisfying for all defect types. A relevant thigh pain was present in 9% of patients, but only 2.4% of the cases demonstrated an unstable stem. Seven stems (2.4%) failed, all occurring in type III defects and in cases with suboptimal canal filling (less than 75%). 

Christie et al. reviewed 129 revision hips at a minimum follow-up of 4 years, revised using a primary cementless implant (S-Rom, DePuy, Warsaw, IN, USA; conical tapered modular stem) [12]. Most of the bone defects were graded I (65%), with grades II and III making up 35% of the cases. The average Harris hip score improved from 47.7 points pre-operation to 87.5 points post-operation. In 11.9% of the hips, a modest thigh pain was reported. Stable bony ingrowth was found in 92.2% of the stems, and 81.4% showed spot welds. Intraoperative fractures occurred in 22.5% of the cases, with no post-operative progression after fixation. The total re-revision rate was 1%, and the aseptic failure rate was 2.9% at a mean follow-up of 6.2 years.

In a case series by Tauber et al., 24 revision hips after failed cemented stems were performed using a conventional standard stem (CLS, Zimmer, Warsaw, IN, USA: extensively coated single wedge tapered stem) [13]. The reason for the revision was aseptic loosening in all cases but one (periprosthetic infection). Early complications occurred in 40.9% of the cases and late complications (mostly leg length discrepancy) occurred in 31.8%. One failure occurred due to recurrent dislocation (stem subsidence) (4.5%), and two poor outcomes (8.3%) were due to intraoperative femoral fractures and subsidence. The authors noticed that the failures were tendentially linked to inadequate cement removal and the loss of collar bone support.

Moreland et al. described 137 hips with Paprosky defects graded I to III, revised using a cementless primary stem (AML, DePuy, Warsaw, IN, USA) [14]. Most of the failed stems were cemented primary implants (63%). At a mean follow-up of 9.3 years, 7% of the stems were revised: half of the cases failed due to aseptic loosening (40% of these had an intraoperative femoral fracture) and half due to periprosthetic infection. In 10% of the cases, a remarkable thigh pain was reported. In 83% of the stems, good osseointegration was detected. In cases with optimal canal filling, 89% of the stems achieved a valid bony ingrowth, whereas only 54% integrated in cases of incomplete canal filling.

El-Deen et al. assessed 54 revision hips mainly performed for failures due to aseptic loosening, which were reimplanted using a primary cementless stem (S-Rom, DePuy, Warsaw, IN, USA) [15]. Most of the revised cases required cemented components (66.7%). The femoral bone defects were graded I (33.3%), II (18.5%), III (31.5%), and IV (1.9%). The Harris hip score improved from 40 (pre-operative) to 80 at 4.5 years. 96% of the implants showed good osseointegration; the other stems subsided but eventually integrated. No stem revisions were performed. 

Kelly et al. assessed 33 stem revisions, mainly performed for aseptic loosening (43%) or infection (22%), that used a cementless primary femoral component (Secur-Fit Plus, Stryker, Kalamazoo, MI, USA: double wedge stem, proximally coated with hydroxyapatite) [16]. All patients had modest bone loss graded AAOS type I, II, or III (segmental, cavitary, and combined defects). Three stems (9%) were revised due to infection at an average follow-up of 5 years. A good bony ingrowth was evident in all the cases. Three intraoperative proximal femoral fractures occurred (9%), requiring cerclage wires. 

Salemyr et al. reported the outcomes of 62 revisions for aseptic loosening in Gustilo and Pasternak bone defects I and II (mostly) that used conventional cementless stems (Bi-Metric, Biomet, Warsaw, IN, USA: proximally hydroxyapatite-coated tapered stem) [17]. Most of the revised stems (85.5%) were cemented, and in 53.2% of the cases, bone grafting was required. For 23 patients (37.1%), an excellent-to-good clinical outcome was achieved at the mid-term, with a mean Harris hip score of 75 points. 85.5% of the stems showed good osseointegration, while 30.6% subsided up to a maximum of 8 mm. 14.5% of the hips dislocated, 6.5% had intraoperative fractures, and 6.5% showed post-operative fractures. Four hips required re-revisions due to fractures or dislocations. At 6 years, the survival rate was 95%. 

Thorey et al. described the clinical and radiographic outcomes of 79 Paprosky I–II revisions performed with a primary cementless stem (Bicontact, Aesculap, Tuttlingen, Germany: ream and broach stem, with a proximal fixation and a trochanteric wing) [18]. The paper was an update of a 2007 article [19]. Most of the revisions were due to aseptic loosening (69, 87.3%), and a third of the implants (29, 36.7%) were cemented. Four re-revisions occurred, half for re-infection and half for aseptic loosening, at a mean follow-up of 6.8 years. The clinical outcomes were reassuring (mean Harris hip score: 78.9 at latest follow-up). A mild stress shielding was evident in Gruen zones I and VII. The 10-year survival rate was 96.2% 

Pineroli et al. investigated the short-term outcomes of 41 first-time revisions that used a cementless primary stem (Corail, DePuy, Warsaw, IN, USA: extensively coated single tapered stem) [20]. 73.2% of the cases were due to aseptic loosening and 63.4% were cemented stems. Almost all the cases were graded I and II according to the Paprosky. The 30-month clinical outcomes were positive (mean Harris hip score of 90 points). Three fractures (10%) occurred intraoperatively, during stem removal. All the stems showed good signs of osseointegration: no stem subsided more than 5 mm. Stem sinking was not different among the three bone defect cohorts. No stem re-revisions were performed. 

Khanuja et al. evaluated 19 revision hips, which mainly failed due to periprosthetic infection, revised with a primary cementless stem (Accolade TMZF, Stryker, Kalamazoo, MI, USA: tapered, proximally porous-coated stem) [21]. The femoral bone loss was graded as Paprosky I in 6 hips (31.6%) and Paprosky II in 13 hips (68.4%). At 5 years, the survival rate was 95%. One revision occurred due to aseptic loosening, but the other implants showed good osseointegration without malalignment or progressive radiolucency. 

Tetreault et al. investigated 277 revision hips in the presence of femoral bone loss graded I to IIIA according to the Paprosky classification system [22]. Conventional fully porous-coated stems were adopted (52%) when a femoral canal was less than 18 mm and a minimum 4 cm scratch fit could be achieved in the femoral canal (as a rule, type I defects with inadequate intraoperative stability and very distal defects are amenable to specific adjustments). Finally, 122 revisions were included. Most of the revisions were due to periprosthetic infections (44%) or aseptic loosening (39%). The Authors noticed that the revision stem did not bypass the tip of the previous failed stem in 78% of the cases (regardless of Paprosky classification). At 2 years of follow-up, the mean Harris hip score was 76 points (range: 21–100). A re-revision was necessary in 4.9% of the cases due to aseptic loosening, preferentially in type II defects and in cases of 19 mm diameters or larger. 

Gastaud et al. reviewed 43 revisions in Paprosky I and IIA defects performed using a cementless primary stem (Linea, Tornier, FR: proximally hydroxyapatite-coated on sand-blasted titanium anatomical stem) [23]. The Harris hip score improved from 58 to 85 points after 4 years. No intraoperative fractures occurred. Dislocations were experienced by 2 patients (4.7%). The survival rate of the implants was 85% at 80 months, with no stem revisions. All the stems integrated, 7% with only a good bony ingrowth. 

Cavagnaro et al. described the outcomes of 84 revision hips performed as a second-stage procedure for periprosthetic hip infections, using two types of cementless primary stems (CLS and Wagner Cone, Zimmer, Warsaw, IN, USA) [24]. The bone defects were graded according to the Paprosky classification as I (44%), II (52.4%), and IIIA (3.6%). 39.3% of the cases required a cortical window for stem extraction. In all the cases, intraoperative stability of the cortical window was achieved during second-stage revision. The Harris hip score significantly improved from 41.7 to 90.8 at a mean follow-up of 37.4 months. The radiographic evaluation showed that all the stems were integrated. The complication rate was 7.1% (not linked to stem). The stem survival rate was 96.3%.

Wood et al. matched 10 revisions performed with primary cementless stem (Corail, DePuy, Warsaw, IN, USA) with 10 revisions performed with revision long stems [25]. The 1-year subjective and objective outcomes were comparable. Operative times and hospital costs were significantly lower for the primary stem cohort (46 min shorter and USD 3707.64 less expensive).

Barakat et al. described 30 revisions (27.7% of all the revisions in a 4-year period) performed using a primary cementless stem (Furlong hydroxyapatite-ceramic (HAC)-coated, Joint Replacement Instrumentation Ltd., Sheffield, UK: extensively coated tapered stem with a lateral fin) [26]. Around half of the revisions (46.7%) were due to metallosis. The bone defects were graded Paprosky I in 60% of the cases, Paprosky II in 30%, and Paprosky IIIA in 10%. At a mean follow-up of 44 months, the survival rate was 100%. The Oxford hip score improved from a pre-operative value of 14 to a score of 35 points in the post-operative setting. No thigh pain was reported. No radiographic signs of loosening were evident. 

Romagnoli et al. assessed the outcomes of 94 revision hips performed for Paprosky I and II femoral defects that used a cementless primary implant (Wagner Cone, Zimmer, Warsaw, IN, USA) at a mean follow-up of 12.7 years [27]. Most of the revisions were due to aseptic loosening (92.6%). Out of the 94 hips, only 70 were finally evaluated. The final Harris Hip score was 86.9 points, with no differences between Paprosky I or II defects. All the stems showed signs of good osseointegration, and no femoral components subsided more than 2 mm. The 10-year survival rate was 95.9%, with three stems revised for infection and periprosthetic fractures.

The long-term outcomes for 41 revision hips performed using a cementless primary implant (Wagner Cone, Zimmer, Warsaw, IN, USA) in Paprosky defects graded II and III were described by Park et al. [28]. Only 28 patients were available at a long-term follow-up. All the cases failed due to aseptic loosening. Most of the cases were graded Paprosky II A (35.7%). At 14.8 years, the mean Harris hip score was 83 points. All the stems were fully integrated. Around 10.7% of the femoral components subsided more than 5 mm. Re-revisions occurred in 14% of the cases (15-year survival rate of 83.4%), but no femoral components were exchanged.

Skibicki et al. evaluated a case series of 70 first-time revision hips for femoral defects graded Paprosky I and II at a mean follow-up of 3 years [29]. A total of 35 different primary cementless stems (single-wedge with a complete hydroxyapatite coating and double-wedge with a partial hydroxyapatite coating) were compared to 35 revision stems (modular and non-modular tapered stems). The two cohorts were homogenous in terms of demographics and pre-operative features, apart from surgical approach. The clinical results were comparable, and tendentially better for conventional stems. One intraoperative femoral fracture occurred in the revision stem cohort, whereas four post-operative complications (dislocations) were traced in the primary stem group and one in the revision stem group. No stem re-revisions were performed. No radiographic differences were observed in terms of subsidence or leg length discrepancy. 

Pai et al. compared two cohorts who received revisions for aseptic loosening in Paprosky I and II defects [30]. The two cohorts included 28 different primary stems and 50 different revision stems. The two groups were comparable in terms of demographics and pre-operative surgical features. The primary stem cohort had a shorter surgical time, lower intraoperative blood loss, and a lower transfusion rate. At a 72-month follow-up, the primary stem cohort showed a lower incidence of complications and a lower rate of intraoperative fracture, but there were no differences in terms of clinical outcomes or survival rates.

In a matched-pair comparison of revisions performed for aseptic loosening for Paprosky I and II bone loss, Tsai et al. described the 7-year outcomes of 24 revisions performed with primary stems (different types) and 72 revisions performed with long revision stems (fully porous-coated components) [31]. In some cases, a cemented revision was performed. There were no significant differences in terms of radiographic subsidence or revision rates. The canal fill ratio was significantly higher in the long stem cohort. 

Willems et al. compared two cohorts of revision hips in Paprosky II bone defects. The two groups were homogenous in terms of pre-operative status and indications for revision (mainly aseptic loosening and secondary infection) [32]. One group was treated using a primary tapered stem (Wagner Cone, Zimmer, Wausau, IN, USA), and the other received a long modular tapered stem (Revision, Lima Corporate, San Daniele del Friuli, Italy). The primary stem cohort achieved a lower complication rate (31% vs. 43.3%) and a lower revision rate (3% vs. 13%). The most relevant reason for revision was subsidence. No differences in terms of clinical outcomes were observed.

Mangin et al. described 35 cases that were treated with a one-stage revision for periprosthetic hip infections using a cementless primary stem (Avenir, Zimmer, Warsaw, IN, USA: hydroxyapatite extensively coated single wedge tapered stem) in Paprosky type 1 defects [33]. 91.4% of the cases resolved the infection at a median follow-up of 5 years. The mean Harris hip score was 77 points. A total of 96.8% of the femoral components showed a good bony ingrowth. 

## 3. Short Stems

Liu et al. described a sizeable cohort of 381 revisions where short stems (Trilock, DePuy, Warsaw, IN, USA) were adopted for femoral defects graded up to Paprosky IIIA. Bone grafting was performed as needed [34]. The mid-term survival rate of revisions using short stems was 94.5%. Multivariate analysis identified three risk factors for short stem failure (re-revision): ageing (HR: 1.056, CI95%: 1.012–1.102), osteoporosis (HR: 2.802, CI95%: 1.097–7.157), and intraoperative periprosthetic femoral fracture (HR: 5.477, CI95%: 2.156–13.913). The risk for short stem failure increased by 5.6% for every year of age increase, and this factor was deeply connected to osteoporosis. Both risk factors were probably not mitigated by bone grafting, showing that secondary fixation of short stems may be compromised by biological factors due to ageing. Intraoperative periprosthetic femoral fractures were found to be the most severe negative prognostic factor: the Authors advised for a prompt stem exchange with long stems with diaphyseal fixation instead of an isolated fracture fixation.

Coutandin et al. reported a small case series of 47 revision hips using cementless femoral components, including 46.8% that were performed using short femoral stems, 27.3% that included resurfacing hip revisions, 45.4% that were revisions of short stems, and 27.3% that were revisions of standard femoral stems using short stems (“downsizing”) [35]. The Authors adopted a meta-diaphyseal anchoring short stem (Optimys, Mathys, Bettlach, Switzerland) as a first option in two cases and as an alternative in four cases. In the first case, the distal cement plug could not be removed and the short stem was implanted to avoid a stem with a more distal fixation. In the second case, after a transfemoral approach, rotational stability with the revision stem could not be achieved and a short stem was adopted. In the fourth case, a short stem was implanted after multiple via falsa with a standard cementless stem. In the sixth case, the sclerotic diaphyseal bone could not be easily reamed and a short stem was chosen. Although in all cases a successful outcome was achieved, the use of short stems in revisions was warranted in very selected cases after a careful evaluation of all the cons. 

## 4. Discussion

With the increasing number of revisions and the widespread use of arthroplasty solutions, even among young patients, the interest in conservative revision approaches for the femoral side are relevant [1,2,3]. The use of a cementless primary stem may provide a solid biological fixation, sparing the more distal bone stock, and reducing the challenges associated with surgery and the preparation of the diaphyseal bone stock [5]. Moreover, many cementless primary stems may provide multiple solutions for proximal biomechanical restoration, even without the use of modularity [5].

**Figure 1:**
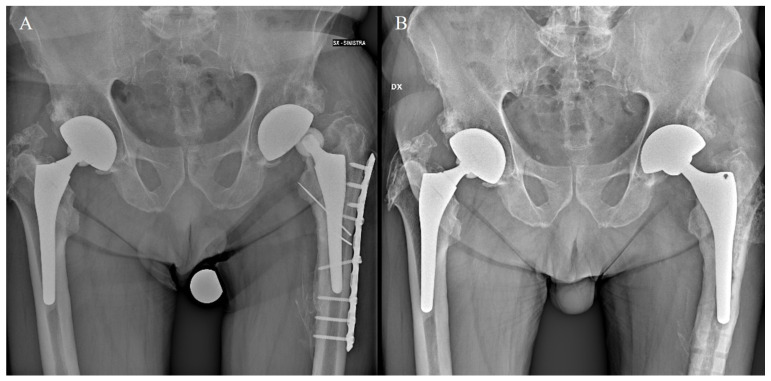
Aseptic loosening of the stem and implant instability occurred after a periprosthetic fracture (**A**). A femoral revision was performed using a cementless primary stem (Apta-fix, Adler Ortho, Milan: extensively coated anatomic stem) implanted in a varus configuration to restore the femoral offset (**B**).

Revisions of the femoral component using cementless primary stems achieved dependable performances in dedicated case series: at mid-to-long-term follow-up, the survival rate was higher than 80%, and the rate of stem aseptic loosening was usually very low or negligible, similar to the survival rates provided in registry studies [36] [Table 1]. Unfortunately, most of the studies were single case series, with no control groups and mixed populations in terms of demographics, revision settings, femoral bone loss, reasons for revision, and sometimes even implants [9,10,11,12,13,14,15,16,17,18,19,20,21,22,23,24,25,26,27,28,29,30,31,32,33]. In the comparative case series, cementless primary stems resulted in lower complication rates (mostly intraoperative fractures), shorter operative times, less blood loss, lower transfusion rates, shorter hospitalization lengths, and even lower costs [25,29,30,31,32]. No differences could be found in terms of clinical outcomes, radiographic assessments (subsidence and leg length restoration) or re-revision rates [25,29,30,31,32]. The varus configuration of the stem is probably more likely to occur with primary stems [25,29,30,31,32] [Figure 1]. 

The indications for the use of primary cementless stems were not always well defined. Tetreault et al. suggested conventional fully porous-coated stems in femoral canals inferior to 18 mm and in cases with a minimum 4 cm scratch fit (amenable to specific adjustments) [22]. However, there are two critical points that the available case series did not enlightened: the femoral bone defects addressable with standard stems and the type of cementless stems suitable for femoral revisions. 

Most of the Authors adopted cementless standard stems for Paprosky defects I and II, and others advised against the use of primary femoral components for severe bone loss (Paprosky III or higher) [Table 1]. As the Paprosky classification is qualitative and does not provide any numerical cut-offs, the use of this classification may be generic and subjective, lacking specificity for revision indications [4,11]. Plus, during revision surgery, the need for further procedures on the femur for stem extraction (cortical window, Wagner transfemoral approach, and extensive trochanteric osteotomy) may impair the femoral bone quality and support, making primary stems an unsuitable choice [5,22]. In a few papers, another relevant factor impairing long-term fixation was canal filling, which could be correlated to the underlying bone defect and possible accessory procedures [9,10,11,14,31]. In general, in cases of canal filling inferior to 80%, an increased risk of suboptimal fixation or even loosening should be expected, mostly if meta-diaphyseal fitting stems are adopted [9,10,11,14,31]. 

The Authors adopted different stem geometries, posing a further confounding factor. In fact, a diaphyseal fitting stem (like Wagner Cone) provides a more distal fixation than a metaphyseal fitting stem (like Corail or CLS), possibly addressing more severe bone loss (some Paprosky IIIA with diaphyseal violation), and extending the indications for primary stems [27].

**Figure 2:**
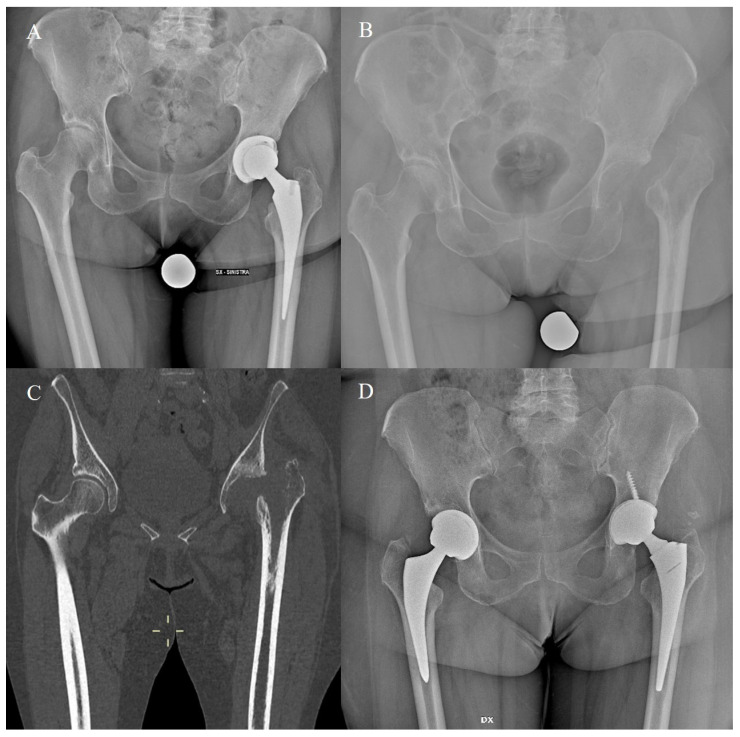
A revision of a primary stem was performed due to periprosthetic hip infection (**A**,**B**). As minimal metaphyseal bone loss was evident (**C**), a cementless primary stem (Hydra, Adler Ortho, Milan: extensively coated single-wedge tapered stem) was adopted, with dependable 5-year outcomes (**D**).

In conclusion, it can be suggested that Paprosky I and II defects can be approached with a standard cementless stem, regardless the stem geometry, whereas Paprosky IIIA defects should be treated with conical tapered stems with more distal fixation (not all these defects are amenable to this treatment; for example, a de-escalation after a revision stem may be hazardous and should be well-planned due to diaphyseal cortical weakening). An intraoperative assessment of the bone defect should always be performed and a revision stem should be available in the operating room [23] [Figure 2].

Revision with a short stem is a controversial procedure, supported by limited clinical evidence from the literature and few expert opinions [34,35,37]. Similar to conventional stems, it is very important to discriminate the correct short stem type and the femoral bone loss. Ideally, many revisions with a supportive proximal femur can be managed with a shortened tapered conventional stem with a meta-diaphyseal anchorage [34]. Short stem revisions could be planned using the Casella’s classification for proximal bone defects in conservative arthroplasties [7]. The classification system considers four determinants: medial neck preservation > 1.5 cm, lateral neck and trochanteric fossa preservation, supportive metaphyseal cancellous bone, and the extent of diaphyseal bone violation < 2 cm [7]. A failed resurfacing arthroplasty leaving a supportive medial neck may be revised even with a neck-retaining short stem [7]. All the other cases may benefit from shortened tapered conventional implants, which can be used even in more severe cases, with a minor diaphyseal bone violation (with a little stem oversizing) [7,34]. A “downsizing” from longer stems should be carefully evaluated and adopted in very selected cases [35,37]. It appears that the possible indications are distal bone/cement plug, retained hardware or bone remodeling in which a femoral osteotomy/transfemoral approach is not feasible/desirable [35,37]. Meta-diaphyseal bone stock should be assessed and bone grafting can be performed if necessary [34]. Stress risers in the presence of thin cortices should be avoided [34,35]. The main contraindications are related to age/osteoporosis and, mostly, intraoperative periprosthetic femoral fractures [34,38].

## 5. Conclusions

Primary cementless stems (in conventional or short lengths) are a promising solution to the need for a conservative revision strategy: Paprosky I and II are the most suitable femoral bone losses to be addressed. However, the correct indications for conservative revisions are still lacking and very hard to define. Currently, very accurate pre-operative planning (which should be confirmed intraoperatively after stem removal) and a consequent wise choice of stem geometry seem to be the cornerstones for a successful revision with a cementless primary stem.

## 6. Future Directions

There is a compelling need for comparative trials on sizeable cohorts in order to determine the correct indications for primary stem usage. In particular, an extensive study about the relationship between stem geometry and femoral bone loss should be performed in order to assess the outcomes of primary stems in specific femoral contexts. To date, the lack of a quantitative femoral bone defect classification is an obstacle to the achievement of this target. 

## Figures and Tables

**Table 1:** Primary stems in revisions: resume of the literature.

Authors	Year of Publication	Number of Hips	Type of Stem	Paprosky Defects	Mean Follow-Up in Months	Re-Revision Rate	Percentage of Integrate Stems
Lawrence et al. [9]	1994	83	AML DePuy	NA	108	20%	89%
Moreland et al. [10]	1995	175	AML DePuy	I, II, III	60	4%	82.8%
Krishnamurthy et al. [11]	1997	297	AML DePuy	I, II, III	99.6	2.4%	82%
Christie et al. [12]	1999	129	S-Rom DePuy	I, II, III	74.4	2.9%	92.2%
Tauber et al. [13]	2000	24	CLS Zimmer	NA	61.9	4.5%	100%
Moreland et al. [14]	2001	137	AML DePuy	I, II, III	111.6	7%	83%
El-Deen et al. [15]	2006	54	S-Rom DePuy	I, II, III, IV	54	0%	96%
Kelly et al. [16]	2006	33	Secur-Fit Plus Stryker	AAOS I, II, III	60	9%	100%
Salemyr et al. [17]	2008	62	Bi-metric Biomet	Gustilo I, II	60	5%	85.5%
Thorey et al. [18]	2008	79	Bicontact Aesculap	I, II	81.6	3.8%	NA
Pineroli et al. [20]	2009	41	Corail DePuy	I, II	30	0%	100%
Khanuja et al. [21]	2014	19	Accolade TMZF Stryker	I, II	49	5%	94.7%
Tetreault et al. [22]	2014	122	Bicontact Aesculap	I, II, IIIA	78	4.9%	85.1%
Gastaud et al. [23]	2016	43	Linea Tornier	I, II	48	15%	100%
Cavagnaro et al. [24]	2019	84	CLS Zimmer Wagner Cone Zimmer	I, II, IIIA	37.4	3.7%	100%
Wood et al. [25]	2019	10	Corail DePuy	I, II, IIIA	12	0%	NA
Barakat et al. [26]	2020	30	Furlong hydroxyapatite-ceramic (HAC)-coated, JRI Ltd.	I, II, IIIA	44	0%	100%
Romagnoli et al. [27]	2020	94	Wagner Cone Zimmer	I, II	152.4	4.1%	100%
Park et al. [28]	2021	297	Wagner Cone Zimmer	I, II, IIIA	177.6	14%	100%
Szibicki et al. [29]	2021	70	Different	I, II	32.4	0%	100%
Pai et al. [30]	2021	28	Different	I, II	75.1	4.8%	NA
Tsai et al. [31]	2022	24	Different	I, II	91.2	4.2%	91.7%
Willems et al. [32]	2022	29	Wagner Cone Zimmer	II	60	3%	93.1%
Mangin et al. [33]	2023	35	Avenir Zimmer	I	60	8.6%	96.8%

## Data Availability

No new data were created or analyzed in this study. Data sharing is not applicable to this article.
